# Consumption of Dark Green Leafy Vegetables Predicts Vitamin A and Iron Intake and Status among Female Small-Scale Farmers in Tanzania

**DOI:** 10.3390/nu11051025

**Published:** 2019-05-07

**Authors:** Wolfgang Stuetz, Victoria Gowele, Joyce Kinabo, Nyamizi Bundala, Hadijah Mbwana, Constance Rybak, Laila Eleraky, Christine Lambert, Hans Konrad Biesalski

**Affiliations:** 1 Institute of Nutritional Sciences, University of Hohenheim, 70599 Stuttgart, Germany; vgowele@sua.ac.tz (V.G.); laila.eleraky@nutres.de (L.E.); christine.lambert@uni-hohenheim.de (C.L.); biesal@uni-hohenheim.de (H.K.B.); 2 Department of Food Technology, Nutrition and Consumer Sciences, Sokoine University of Agriculture, Morogoro 3006, Tanzania; joyce_kinabo@yahoo.com (J.K.); nyamizi80@yahoo.com (N.B.); hadija27@yahoo.com (H.M.); 3 Leibniz Centre for Agricultural Landscape Research (ZALF), 15374 Müncheberg, Germany; constance.rybak@zalf.de

**Keywords:** Dark green leafy vegetables, vitamin A, carotenoids, iron, small-scale farmers, anemia, micronutrient intake, micronutrient status, overweight, Tanzania

## Abstract

Inadequate consumption of micronutrient-dense foods such as vegetables and meat are an important contributing cause for anemia and deficiencies of iron and vitamin A in rural communities of Tanzania. A cross-sectional study was conducted in 2016 to examine nutritional and micronutrient status and their associations to the diet of female small-scale farmers in the sub-humid Kilosa (*n* = 333) and the semi-arid Chamwino (*n* = 333) districts, in the Morogoro and Dodoma region. An overall higher prevalence of overweight (19.7%) and obesity (7.1%) than of underweight (5.9%) was detected. Significantly more women in the two villages of Kilosa (27–40%) than in the two villages of Chamwino district (19–21%) were overweight/obese, but also more frequently had anemia (34–41% vs. 11–17%), iron deficiency (24–32% vs. 15–17%), and low serum retinol (21–24% vs. 8–9%). Overall, only a small proportion of women reached recommended daily micronutrient intakes: 27% for vitamin A, 17% for iron, 7% for zinc, and 12–38% for B-vitamins. The amount of dark green leafy vegetables (DGLV) consumed was the main determinant of vitamin A and iron intake by women in Chamwino and corresponded to higher hemoglobin, serum retinol and iron status than in the villages of the Kilosa district; in agreement, DGLV consumption also predicted iron and vitamin A intake in Kilosa district. DGLV consumed with wholemeal millet was advantageous in terms of women’s vitamin A and iron intake and status over the predominantly maize-rice-based diet lacking vegetables.

## 1. Introduction

Anemia and deficiencies of iron and vitamin A are highly prevalent in rural areas of Africa [1,2,3]. The 2010 Tanzanian Demographic Health Survey covering the whole country, revealed an overall 41% prevalence of anemia (18–64% depending on region), 42% of vitamin A deficiency (37–53% <1.24 µmol/L), and 30% of iron deficiency (7–45% sTfR >8.3 mg/L) among 15–49 year old women [4]. A very recent study in North Eastern Tanzania reported that over one third of women had preconception anemia (36.7%) or iron deficiency (37.6%) [5].

The high prevalence of anemia and deficiency of micronutrients is common in sub-Saharan Africa and is associated with a poor unbalanced diet consisting mainly of cereals, very few animal products and the insufficient micronutrient content of yellow and orange fruits and vegetables [6,7,8]. In addition, persistent infectious diseases such as cholera and malaria increase the risk and prevalence of micronutrient deficiencies. In the Lindi district, Tanzania, nutritional anemia and iron deficiency among children and adults was associated with a monotonous cereal and vegetable-based diet and malaria infection [9].

The consumption of cultivated and wild traditional vegetables could contribute significantly to the micronutrient supply and may be associated with lower prevalence of anemia and MN deficiencies in resource-poor communities [10,11]. The Scale-N project, a multidisciplinary study under the German Federal Ministry of Food and Agriculture (BMEL) call ‘Nutrition-Diversified Agriculture for a Balanced Nutrition in Sub-Saharan Africa’, aimed to achieve food and nutrition security of the rural population in Tanzania by support and development of nutrient sensitive agricultural production, establishment of pocket gardens for production of leafy vegetables and improvement of nutritional behaviour. A baseline study in July 2016 was conducted to determine nutritional and micronutrient status and to identify nutritional gaps among the female small-scale farmers and their school-age children from different villages in the Chamwino and Kilosa district in Dodoma and Morogoro regions, respectively; this included anthropometric and hemoglobin measurements, dietary intake assessment, and blood collection for the analysis of serum micronutrients and infections markers. The baseline survey aimed to identify nutritional gaps in the study population, prior to the planned measures such as training in nutritional behavior and the cultivation of leafy vegetables in home and school gardens. The present study analyzed the micronutrient and nutritional status and their associations with daily food intake of female small-scale farmers (mothers or caregivers) from two different agro-ecological zones with two villages each.

## 2. Materials and Methods

### 2.1. Study Population and Field Procedure

The baseline study of the Scale-N project was carried out in July to August 2016 in the villages Mzula and Chinoje of the Chamwino district, Dodoma, and in the villages Tindiga and Mhenda- Kitunduweta of the Kilosa district, Morogoro. The villagers and study population were practically all self-sufficient small-scale farmers. The semi-arid Chamwino district (350–500 mm annual rainfall) is one of seven districts of the Dodoma region and consists of primarily flat plains; the predominantly sub-humid Kilosa district (600–800 mm annual rainfall) is one of seven districts of the Morogoro region and is characterized by flat plains, highlands, and dry alluvial valleys [12]. According to the 2012 Population and Housing Census, the population of Chamwino District was 330,543, while Kilosa had a population of 438,175 [13].

More than 165 households per village with the inclusion criterion to have a mother and a respective schoolchild at an age between 6–9 years were randomly assigned from the village register. The village- and hamlet leaders and agriculture extension officers of each village supported our field team in the admission of the mother/caregiver-children pairs to the study at the respective schools of the villages. The survey was carried out according to the guidelines laid down in the Declaration of Helsinki and approved by the National Institute for Medical Research and the Ministry of Health, Community Development, Gender, Elderly and Children in Dar es Salaam, Tanzania (NIMR/HQ/R.8a/Vol. IX/2226) and the Ethics Committee Landesärztekammer Baden-Württemberg, Stuttgart, Germany (F-2016-049). Written informed consent was obtained from 669 mothers and/or caregivers for the analysis of nutritional status (anthropometry, dietary intake) and collection of blood for the determination of serum micronutrients. One woman refused to give blood during the study enrolment, one woman did not attend with her own child, and one woman was excluded from the present analysis due to an incomplete questionnaire, giving a total sample size of 666 women within the baseline survey.

### 2.2. Assessment of Maternal Characteristics and Dietary Intake

Height, weight and mid-upper arm circumference (MUAC) were measured for all mother/caregivers using a wooden stadiometer (UNICEF), an electronic scale (Seca 874, Seca GmbH & Co KG Hamburg, Germany) and a standard MUAC tape (UNICEF). Weight was recorded to the nearest 0.1 kg while height and MUAC were measured to the nearest 0.1 cm. Body mass index (BMI) was calculated from weight and height measured at admission; suggested cut-offs for underweight (<18.5 kg/m^2^), overweight (≥25 kg/m^2^), obesity (≥30 kg/m^2^), and low MUAC (≤ 24 cm) for adults were applied according to WHO and FANTA recommendations [14,15,16].

Demographic information (personal data) and dietary intake patterns of the study population were collected using the Scale-N project survey tool. This included a 24-h recall to collect data on the amounts of individual food eaten over the last day. Women’s macro- and micronutrient intake was calculated using the Tanzanian Food Composition Tables [17] of which MNs food values were imported into NutriSurvey software (www.nutrisurvey.de) [18]. The database was completed with the available Kenyan and German data base within the NutriSurvey, and with MN values of the mainly consumed dark green leafy vegetable (DGLV) in Kilosa (Corchorus trilocularis) and Chamwino district (sun dried Ceratotheca sesamoides), both analyzed in Hohenheim using HPLC and ICP-OES techniques [19]. The calculated intakes of macro and micronutrients were compared with recommended dietary intakes [20,21]. Individual assessed food (e.g., grams of eaten papaya or cassava) was further merged to 10 important food groups (millet, maize, rice, DGLV, other vegetables, legumes, roots, fruits, meat, and fish) in order to compare the frequency and amount of food groups consumed between the districts and to elucidate food groups responsible for iron, vitamin A, and zinc intake (using linear regression models).

### 2.3. Blood Collection and Laboratory Analysis

Hemoglobin concentrations of the mothers and caregivers were measured on study site during blood collection, using a drop of venous blood taken from the sterile safety multifly to micro-cuvettes and a portable battery-operated hemoglobinometer (HemoCueHb 201+, Angelhom). Anemia was defined as hemoglobin <120 g/L for non-pregnant and <110 g/L for pregnant women [22]. Whole blood of 3-5 mL was drawn using safety multifly needles and serum-gel monovettes (Vacuurad) which were subsequently centrifuged at 1850xg for 15 min using a REMI C854/4 centrifuge (REMI MOTORS LTD, Mumbai, India). Serum aliquots were distributed in Eppendorf tubes, frozen at −20 °C after daily return from the villages, and at −80 °C at the Sokoine University of Agriculture (SUA) in Morogoro before being sent on dry ice to Stuttgart-Hohenheim and kept frozen at −80 °C until the analysis on fat soluble vitamins and carotenoids using HPLC as previously described [23] with minor modifications. Briefly, serum (40 μL) was extracted with an ethanol/n-butanol mixture containing β-apo-8′-carotenal-methyloxime as an internal standard. After centrifugation, the clear supernatant was analyzed using a Shimadzu HPLC (LC-10AD) equipped with fluorescence- (RF-10Axl, set at EX/EM of 325/470 nm for retinol and of 298/328 nm for α-/γ-tocopherol) and UV-Vis detector (SPD 20A, set at 450 nm). Retinol, tocopherols, and the carotenoids lutein/zeaxanthin, β-cryptoxanthin, lycopene, and α-/β-carotene were separated using a ReproSil 80 ODS-2 column (3 μm, 250 × 4.6 mm; Dr. Maisch GmbH, Ammerbuch, Germany) and an eluent as described previously [24] at a flow rate of 1.5 mL/min. Aliquots of serum were transferred (on dry ice) to the VitMin Lab (Dr. JG Erhardt, Willstaett) in order to be analysed for iron status, infection markers, and serum zinc. In brief, serum ferritin and soluble transferrin receptor (sTfR), as indicators for iron status, and the acute phase proteins C-reactive protein (CRP) and α-1 glycoprotein (AGP) were measured in duplicate by ELISA [25,26], while serum zinc was determined by a sensitive colorimetric assay [27,28]. The intra-assay coefficients of variation (CVs) of a pooled sample (*n* = 10) ranged from 3.7% for ferritin to 5.6% for CRP; inter-assay CVs of the same pool (*n* = 20 different plates) were 5.1% for ferritin, 6.5% for sTfR, 7.1% for CRP and 8.8% for AGP.

Iron deficiency was defined as ferritin <12 µg/L and/or sTfR >8.5 mg/L [29], while total body iron stores (IST) were calculated according to an equation using ferritin and sTfR [30]. Cut-off values of >5 mg/L for CRP and >1 g/L for AGP were used to define an acute phase response by infection or inflammation [31]. Retinol <1.05 and <0.7 µmol/L was considered indicative of low vitamin A status and vitamin A deficiency, respectively [32], while serum zinc <0.66 mg/L was used to indicate low/deficient zinc status [33].

### 2.4. Statistical Analysis

Demographic characteristics, anthropometrics, hemoglobin, biomarkers and micronutrients in serum and the calculated macro- and micronutrients intake of the mothers were described using medians and interquartile ranges (IQR: 25% and 75% percentile) for continuous variables and frequencies (%) for categorical data. Differences of socio-demographic data, micronutrient status and micronutrient intake between the women of the four villages were compared using Kruskal-Wallis-, Mann-Whitney U- and Chi-Square test, as appropriate.

Serum biomarkers, micronutrient and food intakes were transformed to normal distribution using square root (SR) or logarithmic (LN) transformation as appropriate and are described by geometric means with 95% confidence intervals (CI). The influence of factors such as infection markers (CRP) on serum micronutrients were assessed using linear regression analysis (Table A2). Serum micronutrient concentrations between districts (Chamwino vs. Kilosa) were compared using multiple regression analysis adjusted for age, parity, pregnancy status and the acute phase proteins CRP and AGP. Simple linear regression models and crosstabs were used to compare the calculated MN (vitamin A, E, iron, zinc) intake and the prevalence and average amounts of consumed individual foods and food groups between districts. Separate multiple linear regression models with a forward stepwise approach for Chamwino and Kilosa districts were applied to identify the quantitatively relevant consumed food items (derived from the 24-h recall, e.g., grams of leafy vegetables or millet) with a significant contribution (*p* < 0.05) to the calculated iron, vitamin A (RE) and zinc intake. All statistical analyses were conducted using SPSS Version 23; *p* values <0.05 were considered as statistically significant.

## 3. Results

Socio-demographic data of a total of 666 women (mothers and/or caregivers), summarised and separated by the four villages, are given in Table 1. These women had a median age of 36 years, were mainly mothers (84%), of whom 7% were pregnant at the time of the study (Table 1). Women in the different villages were similar regarding total number, age, and proportion of caregivers and pregnancy status. Overall, the prevalence of overweight and obesity was higher than of underweight: 19.7% of all women had a BMI between 25 and 29.9 kg/m^2^, 7.1% of women were obese (≥30 kg/m^2^), while only 5.9% of the women were underweight. Median BMI and MUAC in Tindiga and Mhenda-Kitunduweta, villages of the Kilosa district, were significantly higher than in Mzula and Chinoje, the villages in the Chamwino district; highest prevalence of overweight (27.2%) and obesity (13%) was detected in Tindiga. Approximately, one in four women reported having diarrhoea in the last four weeks, and the same proportion reported having malaria infection in the last 90 days prior to the survey; Mzula was the village with the fewest number of women who reported diarrhea or malaria infection.

Blood biomarkers including infection markers (CRP, AGP), hemoglobin, iron status parameters (ferritin, sTfR), serum retinol (vitamin A), zinc and serum carotenoids and tocopherols are presented in Table 2. The overall prevalence of elevated CRP and AGP was 11% and 12%, respectively, with a slightly higher prevalence of increased AGP and a significantly higher prevalence of increased CRP in Tindiga than in the villages of Chamwino. Hemoglobin concentrations were significantly different between the villages: median hemoglobin was lower in women from Kilosa district and thus anemia prevalence was significantly higher (43% and 36%) than in women from Chamwino (15% and 19%). Accordingly, the iron status in women from Kilosa was also significantly lower than in those from Chamwino; Tindiga was the village with the highest prevalence of iron deficiency and women had the lowest body iron stores (mg iron /kg BW); at the same time these women had the highest BMI and the highest prevalence of overweight and obesity among the four villages. The median serum zinc concentration of the four villages was 0.752 mg/L; the prevalence of low serum zinc (<0.66 mg/L) in Chamwino (23–26%) was higher than in Kilosa villages (13–17%).

Similar to iron, the vitamin A status measured by serum retinol was lower in Kilosa than in Chamwino, thus the proportion of women with low serum retinol (<1.05 µmol/L) was significantly higher in the Kilosa (21–24%) than in the Chamwino villages (8–9%). In addition, the serum carotenoids lutein, zeaxanthin and β-carotene as well as serum α/γ-tocopherol were in Kilosa significantly lower than in Chamwino. In contrast, serum α-carotene was higher in the Kilosa than in the Chamwino villages; the highest median serum β-cryptoxanthin was in Mzula (Chamwino), while serum lycopene was highest in Tindiga (Kilosa). The recalculation of the hemoglobin values and serum parameters of all non-pregnant women confirmed the results of the comparisons in Table 2, with practically the same outcome: women in the Kilosa villages had significantly lower hemoglobin, iron status, serum retinol, α/γ-tocopherol and lower serum β-carotene and lutein-zeaxanthin than those women of the villages in Chamwino (Table A1).

Intake of energy, protein, fat, carbohydrates and of vitamins and minerals assessed by 24 h recalls in the different villages are shown in Table 3. Women of the Kilosa villages reported significantly higher intake of energy, protein, fat and carbohydrates but at the same time significantly lower intake of vitamin A, iron and calcium than those in Chamwino. In contrast, the intake of vitamin E and B vitamins tended to be higher in Kilosa than in Chamwino villages, while that of vitamin C was significantly higher. Mhenda-Kitunduweta (Kilosa) was the village with highest estimated intake of B-vitamins (B1, B6, folate), zinc and magnesium. But overall, only a smaller proportion of women in the various villages reached the recommended daily intakes of the respective micronutrients: 27% for vitamin A, 8% for vitamin E, 12–39% for B-vitamins, 27% for iron, and 7%, 17%, and 54% for zinc, calcium, and magnesium, respectively.

Table 4 summarises the status of micronutrients and the calculated micronutrient intake from the reported amount of food items consumed in the two districts. The higher vitamin A and iron status in Chamwino district is reflected in the correspondingly higher dietary vitamins A (RE) and iron intake. In Kilosa, the higher serum zinc can be explained by the slightly higher zinc intake, whereas the serum tocopherols are significantly lower despite theoretically higher vitamin E (α-TE) in the diet.

The different diets can be summarized as follows: In Chamwino, almost all participants (>90%) reported the consumption of ‘ugali’ (stiff porridge) made from (wholemeal) bulrush millet and dark green leafy vegetables (DGLV), while 66% of women had legumes on the previous day. Other vegetables and foods such as roots, fruits, meat or fish were consumed only by a few women (2–8%) and played a very subordinate role. In Kilosa, almost all women (>90%) reported the consumption of stiff porridge from (polished) maize, 61% women ate rice and 75% legumes, while only 34% of women had DGLV the previous day; further significantly more women in Kilosa than in Chamwino reported the consumption of roots, fruits, meat and fish.

Multiple regression analysis assessing amount and responsible food items for micronutrient intake identified DGLV as the most significant source of vitamin A and iron in Chamwino and of vitamin A in Kilosa district (Table 5). Millet porridge and legumes in Chamwino and legumes, maize porridge, DGLV, rice, and fish in Kilosa were additional key food groups and significant sources for iron and zinc.

## 4. Discussion

The present study confirms the trend of an increasing prevalence of overweight and obesity, combined with an insufficient intake of micronutrients and associated high prevalence of anemia and micronutrient deficiencies in African women of reproductive age from both urban and rural areas [34,35,36]. Although the reported amounts of meals and portion sizes may not reflect the exact caloric intake and obesity problem, significant differences between villages and districts in terms of consumed food, micronutrient intake and respective status was discernible.

The prevalence of overweight, anemia and micronutrient deficiencies in the villages of Chamwino was significantly lower than of those in the Kilosa district, despite the even more monotonous diet. In Chamwino, the main meal consisted of wholemeal millet accompanied by a serving of cooked dark green leafy vegetables (DGLV); the latter are mainly collected locally as indigenous leafy vegetables from the surrounding area, dried in the sun, boiled up with water and other ingredients (e.g., peanuts) and eaten as a vegetable relish called ‘Ilende’ [37]. The high consumption of DGLV in Chamwino was significantly associated with the higher vitamin A and iron intake and status than in Kilosa.

It is noteworthy that DGLV was also identified (via linear regression models) as the main source of vitamin A and iron in the Kilosa villages, where fresh leaves were cooked for the vegetable relish (Table 5). Furthermore, the amount of bioavailable iron and zinc from these DGLV may have been increased by the simultaneous consumption of maize porridge being low in phytate, tannin and polyphenol content [38].

The much higher consumption of DGLV in Chamwino is reflected by the significantly higher serum carotenoids lutein-zeaxanthin and pro-vitamin A active β-carotene; the latter is most likely responsible for the higher serum retinol and thus vitamin A status in Chamwino compared to Kilosa women. An intervention study assessing the impact of DGLV on vitamin A status in women did not yield promising results: de Pee et al. (1995) reported no improvement of vitamin A status by stir-fried DGLV (100-150 g of cassava, water spinach, or spinach containing 3.5 mg β-carotene) as a daily supplement for 12 weeks among lactating women from Indonesia [39]. The authors supposed that β-carotene as pigment-protein complexes located in the chloroplast matrix as well as accompanying carotenoids, fibre and polyphenols in DGLV might have inhibitory effects on β-carotene release and absorption. In contrast, several studies among children and one study among pregnant women showed significant improvements of vitamin A status via leafy vegetables. Cassava and kapok leaves, which were grounded, increased vitamin A status in preschool children in Ghana [40] and a green leafy vegetable powder supplement provided for three months increased hemoglobin and serum retinol in Ghanaian school children [41]. Standardised meals which included provitamin A carotenoids from yellow and green leafy vegetables and fat (daily 4.2 mg of mainly β-carotene and 7 g fat) given five days/week for nine weeks significantly improved total-body vitamin A pool, serum β-carotene and hemoglobin concentrations in Filipino schoolchildren [42]. A study among pregnant Tanzanian women in the third trimester reported a significant association between an increased consumption of green leafy vegetables with oil, which increases bioavailability, and high plasma retinol levels [43]. In the present study, the consumption of DGLV was significantly associated with higher serum β-carotene and retinol. Grinding of sun-dried leafy vegetables to reduce particle size and cooking with oil or groundnuts [44] as is common in the Chamwino villages, as well as the dependence of the population on DGLV as the main source of vitamin A may have been responsible for an increased absorption of provitamin A carotenoids and thus much higher serum retinol and vitamin A status in Chamwino. Our recent analysis on indigenous leafy vegetables collected in the study areas and previous studies confirmed DGLV as excellent sources of pro-vitamin A carotenoids, iron and other minerals [37,45,46,47]. Moreover, the consumption of wholemeal millet with higher iron content vs. polished maize or rice and the influence of β-carotene-rich vegetables on the bio-accessibility of iron from cereals have most likely contributed to the higher iron status in Chamwino women. Amaranth or pure β-carotene significantly enhanced the bio-accessibility of iron and zinc from sorghum in simulated gastrointestinal digestion procedure [48]. The physiological explanation is that β-carotene forms a complex with iron, keeping it soluble in the intestinal lumen and preventing the inhibitory effect of phytates and polyphenols on iron absorption [49,50].

It was remarkable that the women of the two villages in Kilosa had a higher prevalence of overweight and obesity but at the same time a much lower intake of iron and vitamin A (RE) and accordingly a lower hemoglobin, iron status and serum retinol than the women of the Chamwino villages. Even greater amounts of ‘empty calories’ in form of polished maize or rice and too few vegetables are obviously the cause for the more pronounced multiple burden of overweight/obesity, anemia, and MN deficiencies in Kilosa. The unbalanced diet of mainly cereals, especially the high intake of refined maize flour-based stiff porridge (“Ugali”), has already been reported for this area [51]. The authors also showed an insufficient intake of minerals in comparison to recommended daily intakes, and a low food diversity among these farmers despite a rich bio-diversity and food availability (e.g., fruits and vegetables) in Kilosa and neighbouring districts. The dominance of a cereal-based diet with insufficient fruit and vegetable consumption despite the rich local food biodiversity is well known in rural areas of Tanzania [52,53].

An even higher prevalence of overweight (34.6%), obesity (29.6%), anemia (38%; 28%–45% depending on region) and iron deficiency (34% with SF<12 µg/L) than in the present study was reported in a national study among non-pregnant women (*n* = 3089, 15-49 years) from Azerbaijan. Another cross-sectional study among 1530 women of reproductive age from 19 different Vietnamese provinces found a higher prevalence of marginal vitamin A status (19% vs. 7%) in underweight women, but no further relationships of micronutrient status with BMI categories [54]. We found a higher prevalence of anemia among underweight women in the Kilosa villages, but no significant difference in micronutrient status between BMI categories in any of the participating villages (data not presented).

Limitation of the study is the cross-sectional design and single time point of blood biomarkers and dietary intake. Further, under-reporting of portion sizes and of consumed sugar in tea and/or millet porridge as well as of home-brewed beer (from millet, maize or banana) are very likely contributing factors for the too low calculated quantities in consumed calories in a study population with a very high proportion of overweight. The strength of the study includes the large sample size with women from different areas, all enrolled, measured (anthropometry, hemoglobin), and interviewed by the same team within 20 days, and the extensive data set with serum biomarkers measured with established methods in Germany.

## 5. Conclusions

In conclusion, outcomes of the present study suggest a strong impact of dietary habits on the prevalence of overweight and micronutrient deficiencies among Tanzanian female small-scale farmers. In terms of body weight, hemoglobin, vitamin A and iron status, a monotonous but micronutrient-rich diet with wholemeal millet and DGLV was advantageous over a diet consisting mainly of polished maize or rice and insufficient amounts of basically more readily available fruits and DGLV. Introduction of pocket and school gardens to grow Chinese cabbage, Swiss chard, kale and fruits as well as nutritional educational programs, as part of the Scale-N project, are promising measures to reduce high prevalence of anemia and micronutrient deficiencies among small-scale farmers of the study area.

## Figures and Tables

**Table 1:** Characteristics of the study population: mothers and caregivers (CG).

Village	All	Mzula	Chinoje	Tindiga	Mhenda-Kitunduweta	*p*
District		Chamwino		Kilosa		
Mothers/CG, N	666	167	166	169	164	
Age, years ^1^	36 (30, 43)	36 (31, 45)	36 (30, 45)	35 (29, 41)	35 (29, 41)	0.122
Parity, n	4 (3, 6)	4 (3, 6) ^a^	5 (3, 8) ^b^	4 (3, 5) ^c^	4 (3, 6) ^a,c^	<0.001
Mothers, % (n) ^2^	83.6 (557)	79.0 (132)	82.5 (137)	84.6 (143)	88.4 (145)	0.134
Age, mothers, years	34 (29, 40)	33 (30, 42)	35 (29, 41)	34 (28, 40)	34 (29, 39)	0.648
Age, CG, years	53 (43, 62)	57 (49, 65)	53 (46, 65)	48 (39, 59)	54 (39, 60)	0.101
Pregnant, % (n)	7.2 (48)	6.6 (11)	8.4 (14)	8.3 (14)	5.5 (9)	0.685
Weight, kg	53.5 (48.4, 60.8)	52.4 (48.3, 60.1)	53.6 (48.3, 60.3)	55.2 (48.3, 65.0)	52.9 (48.7, 59.5)	0.087
Height, cm	154.6 (151, 158)	155.3 (151, 159) ^a^	156.0 (152, 160) ^a^	153.5 (150, 157) ^b^	153.0 (149, 156) ^b^	<0.001
BMI, kg/m^2^	22.3 (20.5, 25.1)	21.9 (20.1, 24.2) ^a^	21.8 (20.3, 24.1) ^a^	23.4 (20.6, 27.8) ^b^	22.4 (20.7, 25.1) ^b^	<0.001
BMI <18.5, % (n)	5.9 (39)	9.0 (15)	4.8 (8)	5.9 (10)	3.7 (6)	<0.001
BMI, 18.5–24.9, % (n)	67.4 (449)	70.1 (117)	76.5 (127)	53.8 (91)	69.5 (114)	
BMI, 25–29.9, % (n)	19.7 (131)	16.8 (28)	15.1 (25)	27.2 (46)	19.5 (32)	
BMI ≥ 30, % (n)	7.1 (47)	4.2 (7)	3.6 (6)	13.0 (22)	7.3 (12)	
MUAC, cm	27.5 (25.5, 29.8)	27.1 (25.0, 29.0) ^a^	26.9 (25.2, 29.2) ^a^	28.6 (26.0, 32.5) ^b^	27.9 (26.0, 29.5) ^c^	<0.001
MUAC <24.0 cm	7.5 (50)	12 (20)	6 (10)	4.1 (7)	7.9 (13)	0.043
Diarrhoea, 4 weeks, % (n)	23.6 (157)	16.2 (27)	25.9 (43)	20.7 (35)	31.7 (52)	0.006
Malaria, 90 days, % (n)	26.1 (174)	15.0 (25)	28.9 (48)	28.4 (48)	32.3 (53)	0.002

Data are median (interquartile range) ^1^ and percentage (number) ^2^, all such values; *p* values, Kruskal Wallis- and Chi Square (prevalence) test, as appropriate; differences between individual villages were assessed by Mann-Whitney U test and Chi Square test; values within a row not sharing a common superscript letter ^(a,b,c,d)^ are significantly different at *p* < 0.05. BMI, body mass index; MUAC, mid-upper arm circumference; Diarrhoea, reported in the last 4 weeks; Malaria, reported in the last 90 days.

**Table 2:** Hemoglobin, infection- (CRP, AGP) and iron status markers (ferritin, sTfR), and serum micronutrients in mothers/CG *.

Village	All	Mzula	Chinoje	Tindiga	Mhenda-Kitunduweta	*p*
District		Chamwino		Kilosa		
Mothers/CG, N	666	167	166	169	164	
Hemoglobin (g/L) ^1^	127 (118, 135)	134 (126, 142) ^a^	128 (122, 135) ^b^	123 (112, 130) ^c^	124 (114, 131) ^c^	<0.001
Anemia, % (n) ^2^	26.0 (173)	11.4 (19) ^a^	16.9 (28) ^a^	41.4 (70) ^b^	34.1 (56) ^b^	<0.001
CRP >5 mg/L, % (n)	11.4 (76)	8.4 (14) ^a^	9.0 (15) ^a^	16.6 (28) ^b^	11.6 (19) ^a,b^	0.075
AGP > 1g/L, % (n)	12.2 (81)	9.0 (15)	12.7 (21)	14.2 (24)	12.8 (21)	0.505
Ferritin (µg/L)	48.7 (31.7, 82.1)	52.5 (33.1, 91.2) ^a^	57.6 (37.5, 98.1) ^a^	39.9 (22.6, 57.0) ^b^	53.6 (32.8, 80.1) ^a^	<0.001
sTfR (mg/L)	6.56 (5.53, 8.21)	6.49 (5.54, 7.74) ^a^	6.08 (5.12, 7.53) ^a^	7.20 (5.82, 9.72) ^b^	6.60 (5.60, 8.40) ^c^	<0.001
Iron deficiency, % (n)	22.1 (147)	17.4 (29) ^a,c^	15.1 (25) ^a^	32.0 (54) ^b^	23.8 (39) ^b,c^	0.001
IST (mg/kg BW)	5.73 (3.94, 7.79)	6.21 (4.09, 8.22) ^a^	6.51 (4.69, 8.25) ^a^	4.60 (2.19, 6.55) ^b^	5.83 (4.29, 7.48) ^a^	<0.001
Zinc (mg/L)	0.752 (0.68, 0.84)	0.723 (0.65,0.80) ^a^	0.759 (0.68, 0.85) ^b^	0.781 (0.69, 0.84) ^b^	0.767 (0.69, 0.85) ^b^	<0.001
Zinc <0.66 mg/L, % (n)	19.8 (132)	26.3 (44) ^a^	22.9 (38) ^a,b^	16.6 (28) ^b,c^	13.4 (22) ^c^	0.013
Retinol, µmol/L	1.459 (1.15, 1.84)	1.744 (1.34, 2.11) ^a^	1.632 (1.29, 1.92) ^b^	1.268 (1.07, 1.52) ^c^	1.306 (1.08, 1.67) ^c^	0.001
<0.7 µmol/L, % (n)	2.1 (14)	1.2 (2)	2.4 (4)	3.6 (6)	1.2 (2)	0.379
<1.05 µmol/L, % (n)	15.5 (103)	9.0 (15)	7.8 (13)	24.3 (41)	20.7 (34)	<0.001
γ-Tocopherol, µmol/L	0.836 (0.41, 1.56)	1.562 (1.05, 2.04) ^a^	1.376 (0.92, 2.12) ^a^	0.441 (0.28, 0.74) ^b^	0.405 (0.27, 0.64) ^b^	<0.001
α-Tocopherol, µmol/L	19.25 (15.9, 23.7)	24.09 (20.2, 29.2) ^a^	19.18 (16.2, 23.9) ^b^	18.19 (15.2, 21.7) ^c^	17.27 (14.0, 20.2) ^d^	<0.001
α-Carotene, µmol/L	0.119 (0.05, 0.26)	0.063 (0.04, 0.13) ^a^	0.049 (0.03, 0.07) ^b^	0.197 (0.13, 0.32) ^c^	0.260 (0.16, 0.37) ^d^	<0.001
β-Carotene, µmol/L	0.626 (0.40, 0.69)	0.729 (0.46, 1.11) ^a^	0.816 (0.57, 1.09) ^a^	0.498 (0.31, 0.82) ^b^	0.519 (0.34, 0.80) ^b^	<0.001
β-Cryptoxanthin, µmol/L	0.107 (0.05, 0.26)	0.257 (0.14, 0.49) ^a^	0.069 (0.05, 0.11) ^b^	0.065 (0.04, 0.13) ^b^	0.146 (0.07, 0.30) ^c^	<0.001
Lutein/zeaxanthin, µmol/L	1.125 (0.78, 1.60)	1.580 (1.10, 1.93) ^a^	1.456 (1.14, 1.84) ^a^	0.919 (0.65, 1.16) ^b^	0.826 (0.62, 1.10) ^b^	<0.001
Lycopene, µmol/L	0.462 (0.26, 0.69)	0.409 (0.26, 0.59) ^a^	0.351 (0.20, 0.54) ^b^	0.666 (0.45, 0.91) ^c^	0.471 (0.22, 0.71) ^a^	<0.001

* Data are median (interquartile range) ^1^, and percentage (number) ^2^, all such values; *p*-values, Kruskal-Wallis and Chi-Square test, as appropriate. CG, caregivers. Differences between individual villages were assessed by Mann-Whitney U test and Chi-square tests: values within a row not sharing a common superscript letter (^a,b,c,d^) are significantly different at *p* < 0.05; Iron deficiency is defined as ferritin <12 µg/L and/or sTfR >8.5 mg/L; IST, total body iron stores, an equation using ferritin and sTfR [30].

**Table 3:** Reported macro- and micronutrient intake of mothers and caregivers by villages in comparison to recommended nutrient intakes (RNI).

Village	All	Mzula	Chinoje	Tindiga	Mhenda-Kitunduweta	
District		Chamwino		Kilosa		
N	666	167	166	169	164	RNI/day
Energy (Kcal) ^1^	1304 (864, 1824)	996 ^a^ (660, 1360)	873 ^a^ (598, 1196)	1638 ^b^ (1248, 2067)	1753 ^c^ (1429, 2302)	2000–2350
EN ≥ RNI, % (n) ^2^	16.2 (108)	7.8 (13) ^a^	4.2 (7) ^a^	23.7 (40) ^b^	29.3 (48) ^b^	
Protein (g)	30 (18, 47)	22 (14, 35) ^a^	18 (14, 29) ^b^	36 (26, 52) ^c^	48 (34, 67) ^d^	47–55
Pro ≥ RNI, % (n)	23.4 (156)	12.6 (21) ^a^	2.4 (4) ^b^	28.4 (48) ^c^	50.6 (83) ^d^	
Fat (g)	24 (13, 41)	18 (10, 29) ^a^	11 (6, 19) ^b^	37 (25, 52) ^c^	35 (22, 51) ^c^	30-35%
EN by fat (%)	18 (12, 24)	21 (15, 27) ^a^	14 (10, 22) ^b^	20 (14, 26) ^a^	17 (12, 22) ^c^	
Carbohydrates (g)	204 (121, 314)	134 (83, 192) ^a^	117 (79, 157) ^b^	296 (217, 365) ^c^	307 (249, 387) ^d^	
EN by CHO (%)	71 (64, 76)	67 (59, 75) ^a^	74 (67, 76) ^b^	71 (65, 76) ^b^	72 (66, 77) ^b^	>50%
Fiber (g)	27 (20, 39)	28 (22, 39) ^a^	32 (24, 48) ^b^	21 (15, 30) ^c^	29 (23, 43) ^a,b^	>30g
PUFA (g)	4.0 (0, 11)	9.4 (5.5, 11.7) ^a^	12.4 (10.9, 22) ^b^	0 (0, 0.3) ^c^	0.1 (0, 0.7) ^d^	
Vitamin A (μg)	269 (108, 604)	309 (173, 599) ^a^	381 (226, 670) ^b^	209 (41, 693) ^c^	132 (36, 336) ^d^	500, 800
RE ≥ RNI, % (n)	27.5 (183)	28.1 (47) ^a^	36.7 (61) ^a^	29.0 (49) ^a^	15.9 (26) ^b^	
Vitamin E (mg)	1.2 (0.2, 2.3)	1.6 (0.4, 3.3) ^a^	0.1 (0, 1) ^b^	1.3 (0.8, 2.3) ^a^	1.8 (1.1, 3.4) ^c^	7.5
α-TE ≥ RNI, % (n)	8.1 (54)	9.0 (15) ^a^	3.0 (5) ^b^	9.5 (16) ^a^	11.0 (18) ^a^	
Vitamin B1 (mg)	0.8 (0.5, 1.1)	0.7 (0.5, 1) ^a^	0.7 (0.5, 1) ^a^	0.7 (0.4, 1) ^a^	1.0 (0.8, 1.3) ^b^	1.1, 1.4
B1 ≥ RNI, % (n)	23.3 (155)	19.8 (33) ^a^	18.7 (31) ^a^	16.0 (27) ^a^	39.0 (64) ^b^	
Vitamin B2 (mg)	0.8 (0.5, 1.1)	0.7 (0.5, 1) ^a^	0.9 (0.7, 1.3) ^b^	0.6 (0.4, 0.9) ^c^	0.8 (0.6, 1.2) ^a^	1.1, 1.4
B2 ≥ RNI, % (n)	23.4 (156)	16.8 (28) ^a^	34.3 (57) ^b^	15.4 (26) ^a^	27.4 (45) ^b^	
Vitamin B6 (mg)	1.1 (0.8, 1.6)	0.9 (0.7, 1.3) ^a^	0.9 (0.7, 1.5) ^a^	1.1 (0.9, 1.6) ^b^	1.4 (1.2, 1.8) ^c^	1.3-1.9
B6 ≥ RNI, % (n)	38.6 (257)	24.6 (41) ^a^	33.7 (56) ^a,b^	36.7 (62) ^b^	59.8 (98) ^c^	
B12 (µg) ^1^	0 (0, 0.4)	0 (0, 0) ^a^	0 (0, 0) ^a^	0 (0, 0.8) ^b^	0 (0, 2.3) ^b^	2.4, 2.6
B12 ≥ RNI, % (n)	12.0 (80)	3.0 (5) ^a^	0.6 (1) ^a^	21.3 (36) ^b^	23.2 (38) ^b^	
Folic acid (μg)	283 (183, 441)	246 (171, 379) ^a^	251 (180, 383) ^a^	281 (144, 392) ^a^	396 (269, 587) ^b^	400, 600
FA ≥ RDI, % (n)	27.9 (186)	21.6 (36) ^a^	22.3 (37) ^a^	22.5 (38) ^a^	45.7 (75) ^b^	
Ascorbic acid (mg)	12.0 (2.2, 41.6)	5.6 (1.1, 18.8) ^a^	1.9 (1.0, 9.5) ^a^	24.5 (9.4, 53.0) ^b^	37.2 (14.1, 67.1) ^c^	45, 55
AA ≥ RNI, % (n)	22.5 (150)	13.2 (22) ^a^	7.2 (12) ^a^	30.2 (51) ^b^	39.6 (65) ^b^	
Iron (mg)	17.3 (10.8, 26.9)	20.9 (15.2, 30.1) ^a^	28.8 (21.3, 41) ^b^	10.8 (7.5, 14.3) ^c^	13.2 (9.6, 17.9) ^d^	11–29
Iron ≥ RNI, % (n)	27.0 (180)	37.7 (63) ^a^	53.6 (89) ^b^	8.3 (14) ^c^	8.5 (14) ^c^	
Zinc (mg)	7.0 (5.2, 9.9)	6.4 (4.5, 8.9) ^a^	7.2 (5.5, 11.5) ^b^	6.6 (5.0, 9.1) ^a^	8.0 (6.0, 10.9) ^b^	9.8–14
Zinc ≥ RNI, % (n)	24.3 (162)	19.8 (33) ^a,b^	28.9 (48) ^b,c^	17.8 (30) ^a^	31.1 (51) ^c^	
Calcium (mg)	463 (195, 879)	608 (350, 919) ^a^	777 (515, 1207) ^b^	203 (97, 469) ^c^	254 (145, 578) ^d^	1000–1200
Ca ≥ RNI, % (n)	17.3 (115)	14.4 (24) ^a^	28.9 (48) ^b^	9.5 (16) ^a^	16.5 (27) ^a^	
Magnesium (mg)	233 (149, 338)	240 (143, 379) ^a^	228 (149, 356) ^a^	187 (127, 285) ^b^	265 (183, 354) ^c^	220
Mg ≥ RNI, % (n)	53.6 (90)	53.9 (90) ^a^	53.0 (88) ^a^	43.2 (72) ^a^	64.6 (106) ^b^	

Figures are median (interquartile range - IQR) ^1^ and percentage (number) ^2^, all such values. RE, retinol equivalent; α-TE, α-tocopherol equivalent (= vitamin E equivalent). RNI /day: Recommended daily nutrient intake of macro- and micronutrients adjusted for age (19-50, 51-65 y), gender and pregnancy status following the D-A-C-H [20] and FAO/WHO [21] recommendation; for zinc the low bioavailability and for iron the 10% bioavailability was applied [21]. *p* values: Kruskal-Wallis test for continuous variables and Chi-square test for prevalence; all *p* values are < 0.001, except for EE ≥ RDI = 0.042, Zinc ≥ RDI = 0.008 and Mg ≥ RDI = 0.002; villages not sharing a superscript letter ^(a,b,c,d)^ are significantly different (*p* < 0.05).

**Table 4:** Micronutrient status and micronutrient and food intake in women of Chamwino vs. Kilosa district.

MN status	Chamwino, *n* = 333		Kilosa, *n* = 333		Beta
Retinol, µmol/L	1.560 (1.49, 1.64)		1.227 (1.12, 1.34)		0.350 **
β-Carotene, µmol/L	0.619 (0.56, 0.68)		0.437 (0.36, 0.52)		0.257 **
Lutein zeaxanthin, µmol/L	1.550 (1.47, 1.63)		0.941 (0.83, 1.06)		0.532 **
γ-Tocopherol, µmol/L	0.918 (0.84, 1.00)		0.298 (0.25, 0.35)		0.677 **
α-Tocopherol, µmol/L	16.41 (15.7, 17.1)		13.36 (12.4, 14.4)		0.342 **
Iron status (mg/kg BW)	4.836 (4.47, 5.23)		3.944 (3.33, 4.60)		0.188 **
Zinc, mg/L	0.747 (0.73, 0.76)		0.778 (0.74, 0.81)		−0.131 *
**MN intake**					
RE intake, µg	422 (356, 493)		232 (198, 269)		0.236 **
EE intake, mg	1.03 (0.75, 1.36)		2.10 (1.81, 2.42)		−0.215 **
Iron intake, mg	24.3 (22.2, 26.6)		11.9 (11.2, 12.8)		0.512 **
Zinc intake, mg	6.97 (6.46, 7.52)		7.18 (6.81, 7.58)		−0.030
**Food intake**	% (N)	Grams	% (N)	Grams	
Millet (porridge)	93 (311) **	581 (385, 875)	2 (8)	195 (130, 293)	0.282 **
Maize (porridge)	10 (33)	244 (195, 306)	91 (304) **	408 (380, 437)	−0.239 **
Rice (with oil) ^1^	3 (11)	339 (255, 449)	61 (202) **	343 (322, 366)	−0.006
Banana (cooked)	0.3 (1)	35 (7, 175)	21 (71)	152 (126, 184)	−0.213 *
DGLV ^2^	92 (308) **	190 (166, 218) **	34 (114)	131 (116, 147)	0.253 **
Vegetables ^3^	8 (26)	99 (71, 137)	29 (97) **	126 (109, 147)	−0.135
Legumes ^4^	66 (186)	37 (31, 45)	75 (249) **	190 (168, 215)	−0.631 **
Roots ^5^	3 (10)	134 (92, 195)	28 (92) **	209 (186, 236)	−0.231 *
Fruits ^6^	7 (23)	122 (80, 185)	19 (64) **	193 (156, 240)	−0.232 *
Meat ^7^	4 (15)	64 (42, 98)	13 (45) **	78 (63, 97)	−0.122
Fish ^8^	2 (6)	55 (34, 89)	26 (87) **	149 (131, 168)	−0.394 **

The data are geometric mean values (95% CI), calculated from linear regression models or percentages (%) and number (N); * *p* < 0.05; ** *p* < 0.001, calculated via linear regression or Chi-square test. Beta, standardized beta-coefficient of Chamwino vs. Kilosa from simple- (MN- and food intake) and multiple linear regression analysis (MN status adjusted for age, parity, pregnancy, and CRP > 5 mg/L and AGP > 1 g/L). ^1^ Rice cooked with coconut or oil and onions; ^2^ DGLV, dark green leafy vegetables include ‘Mlenda mgunda’ (*Corchorus trilocularis*), ‘Ilende’ (*Ceratotheca sesamoides*), and amaranth-, cow pea-, sweet potato- and pumpkin leaves or spinach; ^3^ Vegetables: okra, pumpkin, tomato, African eggplant, Chinese or white cabbage; ^4^ Legumes include beans, peas, bambara nut and ground nut; ^5^ Roots include cassava, potatoes, yams; ^6^ Fruits include banana, baobab, guava, mango, papaya, water melon; ^7^ Meat include beef, goat, chicken and pork; ^8^ Fish include fish relish and dried sardines.

**Table 5:** Multiple linear regression assessing food consumption (in grams) as predictors for the calculated vitamin A (RE), iron-, and zinc intake in the districts.

Chamwino, *n* = 333				Kilosa, *n* = 333			
(SR) **RE intake**, µg	B	Beta	% RE	(SR) **RE intake**, µg	B	Beta	% RE
(Constant)	9.989460			(Constant)	6.682976		
DGLV (g)	0.047291	0.674 **	92.4	DGLV (g)	0.094123	0.803 **	59.0
Fruits (g)	0.015809	0.105 *	2.7	Banana, cooked (g)	0.025342	0.238 **	11.3
Roots (g)	0.030820	0.094 *	1.7	Vegetables (g)	0.022565	0.186 **	12.6
Vegetables (g)	0.021598	0.086 *	3.2	Fish (g)	0.015515	0.121 **	10.1
				Fruits (g)	0.010694	0.120 **	7.0
(LN) **Iron intake**, mg	B	Beta	% Iron	(LN) **Iron intake**, mg	B	Beta	% Iron
(Constant)	2.294511			(Constant)	1.675986		
DGLV (g)	0.002536	0.604 **	44.9	Maize porridge (g)	0.000670	0.324 **	35.9
Millet porridge (g)	0.000517	0.348 **	37.8	Legumes (g)	0.001831	0.470 **	31.8
Legumes (g)	0.000746	0.122 **	17.3	DGLV (g)	0.001928	0.331 **	13.2
				Roots (g)	0.000813	0.177 **	9.8
				Fish (g)	0.001128	0.177 **	9.3
(LN) **Zinc intake**, mg	B	Beta	% Zinc	(LN) **Zinc intake**, mg	B	Beta	% Zinc
(Constant)	1.126724			(Constant)	1.090365		
Millet porridge (g)	0.000711	0.551 **	42.1	Legumes, all (g)	0.001492	0.476 **	26.0
Legumes (g)	0.001502	0.282 **	17.6	Fish (g)	0.002175	0.424 **	9.4
DGLV (g)	0.001274	0.350 **	35.0	Maize porridge (g)	0.000738	0.444 **	32.4
Fish (g)	0.005122	0.131 **	0.7	Rice (g)	0.000625	0.327 **	19.8
Meat (g)	0.002144	0.135 **	1.4	Roots (g)	0.000712	0.192 **	8.4
Maize porridge (g)	0.000272	0.078 *	3.2	Meat (g)	0.001631	0.162 **	4.0

SR, square root; LN, log-normal; B, beta coefficient; Beta, standardized beta coefficient; * *p* < 0.05, ** *p* < 0.001 (probability of F: entry 0.01, removal 0.05). %, percentage MN contribution of consumed food or food group calculated by frequency and mean consumption according to Table 4. Chamwino: RE intake: R^2^ = 0.425 (R = 0.652); Iron intake: R^2^ = 0.694 (R = 0.833), Zink intake: R^2^ = 0.660 (R = 0.812); Kilosa: RE intake: R^2^ = 0.728 (R = 0.853), Iron intake: R^2^ = 0.514 (R = 0.717), Zinc intake: R^2^ = 0.694 (R = 0.833).

## Appendix A

**Table A1:** Hemoglobin, infection- (CRP, AGP) and iron status markers (ferritin, sTfR), and serum micronutrients in non-pregnant mothers/CG *.

Village	All	Mzula	Chinoje	Tindiga	Mhenda-Kitunduweta	*p*
District		Chamwino		Kilosa		
Mothers/CG, N	618	156	152	155	155	
Hemoglobin (g/L) ^1^	128 (119, 135)	134 (127, 142) ^a^	129 (123, 135) ^b^	124 (114, 130) ^c^	125 (114, 133) ^c^	<0.001
Anemia, % (n) ^2^	25.2 (156)	10.9 (17) ^a^	15.8 (24) ^a^	40.6 (63) ^b^	33.5 (52) ^b^	<0.001
CRP >5 mg/L, % (n)	10.0 (62)	7.1 (11) ^a^	8.6 (13) ^a^	12.9 (20) ^a^	11.6 (18) ^a^	0.291
AGP > 1g/L, % (n)	12.6 (78)	9.6 (15) ^a^	13.2 (20) ^a^	14.2 (22) ^a^	13.5 (21) ^a^	0.620
Ferritin (µg/L)	51.3 (33.6, 83.6)	55.0 (33.6, 91.6) ^a^	59.5 (39.4, 100.3) ^a^	41.9 (25.9, 59.9) ^b^	54.2 (34.4, 80.2) ^c^	<0.001
sTfR (mg/L)	6.58 (5.53, 8.21)	6.50 (5.55, 7.66) ^a^	6.13 (5.26, 7.57) ^a^	7.20 (5.84, 9.82) ^b^	6.55 (5.59, 8.43) ^b^	<0.001
Iron deficiency, % (n)	22.3 (138)	16.7 (26) ^a^	15.8 (24) ^a^	32.9 (51) ^b^	23.9 (37) ^a,b^	0.001
IST (mg/kg BW)	5.84 (4.10, 7.87)	6.29 (4.21, 8.31) ^a^	6.75 (4.91, 8.31) ^a^	4.88 (2.35, 6.71) ^b^	5.85 (4.43, 7.49) ^c^	<0.001
Zinc (mg/L)	0.767 (0.68, 0.84)	0.730 (0.67, 0.80) ^a^	0.767 (0.68, 0.87) ^b^	0.781 (0.69, 0.85) ^b^	0.781 (0.71, 0.89) ^b^	<0.001
Zinc <0.66 mg/L, % (n)	17.6 (109)	23.7 (37) ^a^	19.1 (29) ^a,b^	14.2 (22)^b^	13.5 (21) ^b^	0.065
Retinol, µmol/L	1.497 (1.18, 1.87)	1.757 (1.39, 2.16) ^a^	1.645 (1.33, 1.92) ^b^	1.297 (1.09, 1.55) ^c^	1.329 (1.10, 1.70) ^c^	0.001
<0.7 µmol/L, % (n)	1.5 (9)	0.6 (1) ^a^	1.3 (2) ^a^	2.6 (4) ^a^	1.3 (2) ^a^	0.546
<1.05 µmol/L, % (n)	13.8 (85)	7.7 (12) ^a^	5.9 (9) ^a^	21.9 (34) ^b^	19.4 (30) ^b^	<0.001
γ-Tocopherol, µmol/L	0.853 (0.42, 1.63)	1.638 (1.07, 2.07) ^a^	1.398 (0.96, 2.18) ^a^	0.469 (0.28, 0.75) ^b^	0.425 (0.27, 0.67) ^b^	<0.001
α-Tocopherol, µmol/L	18.93 (15.6, 23.4)	24.14 (20.2, 29.5) ^a^	19.18 (16.2, 23.7) ^b^	17.72 (15.2, 21.0) ^c^	16.83 (13.9, 19.7) ^d^	<0.001
α-Carotene, µmol/L	0.113 (0.05, 0.25)	0.063 (0.04, 0.12) ^a^	0.049 (0.03, 0.07) ^b^	0.186 (0.12, 0.29) ^c^	0.259 (0.16, 0.37) ^d^	<0.001
β-Carotene, µmol/L	0.621 (0.40, 0.99)	0.732 (0.46, 1.11) ^a^	0.816 (0.58, 1.10) ^a^	0.471 (0.30, 0.80) ^b^	0.516 (0.34, 0.80) ^b^	<0.001
β-Cryptoxanthin, µmol/L	0.104 (0.05, 0.25)	0.254 (0.14, 0.49) ^a^	0.069 (0.05, 0.10) ^b^	0.061 (0.04, 0.12) ^c^	0.135 (0.07, 0.30) ^d^	<0.001
Lutein/zeaxanthin, µmol/L	1.128 (0.77, 1.60)	1.591 (1.09, 1.93) ^a^	1.456 (1.14, 1.86) ^a^	0.870 (0.64, 1.16) ^b^	0.825 (0.62, 1.10) ^b^	<0.001
Lycopene, µmol/L	0.455 (0.26, 0.68)	0.404 (0.26, 0.59) ^a^	0.351 (0.19, 0.53) ^b^	0.660 (0.44, 0.86) ^c^	0.471 (0.22, 0.71) ^a^	<0.001

* Data are median (interquartile range) ^1^, and percentage (number) ^2^, all such values; *p*-values, Kruskal-Wallis and Chi-Square test, as appropriate. CG, caregivers. Differences between individual villages were assessed by Mann-Whitney U test and Chi-square tests: values within a row not sharing a common superscript letter (^a,b,c^) are significantly different at *p* < 0.05; Iron deficiency is defined as ferritin < 12 µg/L and/or sTfR > 8.5 mg/L; IST, total body iron stores, an equation using ferritin and sTfR [30].

**Table A2:** Determinants of serum micronutrient status marker.

MN Status Marker	B	SE	Beta	*p*
(LN) **Retinol**, µmol/L				
RE intake, µg	5.035×10^-5^	0.000	0.085	0.028
Age, years	0.004	0.001	0.139	<0.001
Parity, n	0.019	0.005	0.132	0.001
Pregnant (=1)	−0.291	0.050	−0.219	<0.001
CRP > 5 mg/L	−0.146	0.042	−0.134	0.001
AGP > 1 g/L	−0.100	0.041	−0.095	0.014
Chamwino (=1) vs. Kilosa (=0)	0.248	0.025	0.359	<0.001
(LN) **β-Carotene**, µmol/L				
Age, years	0.006	0.002	0.098	0.011
Parity, n	0.012	0.011	0.042	0.274
Pregnant =1	0.016	0.102	0.006	0.876
CRP > 5 mg/L	−0.327	0.082	−0.153	<0.001
AGP > 1 g/L	−0.215	0.080	−0.103	0.008
Chamwino (=1) vs. Kilosa (=0)	0.359	0.051	0.265	<0.001
(SR) **Lutein-zeaxanthin**, µmol/L				
Age, years	0.001	0.001	0.033	0.397
Parity, n	0.011	0.004	0.107	0.006
Pregnant (=1)	0.034	0.039	0.034	0.383
CRP > 5 mg/L	−0.160	0.031	−0.197	<0.001
AGP > 1 g/L	−0.107	0.030	−0.135	<0.001
Chamwino (=1) vs. Kilosa (=0)	0.281	0.017	0.545	<0.001
(LN) **α-Tocopherol**, µmol/L				
α−TE intake, mg	0.002	0.003	0.031	0.421
Age, years	0.007	0.001	0.268	<0.001
Parity, n	0.017	0.005	0.141	<0.001
Pregnant (=1)	0.149	0.045	0.129	0.001
CRP >5 mg/L	0.018	0.037	0.019	0.618
AGP >1 g/L	0.008	0.036	0.008	0.833
Chamwino (=1) vs. Kilosa (=0)	0.214	0.022	0.358	<0.001
(LN) **γ-Tocopherol**, µmol/L				
Age, years	0.018	0.003	0.237	<0.001
Parity, n	0.100	0.013	0.294	<0.001
Pregnant (=1)	−0.366	0.124	−0.133	0.003
CRP > 5 mg/L	−0.168	0.102	−0.064	0.099
AGP > 1 g/L	0.284	0.098	0.111	0.004
Chamwino (=1) vs. Kilosa (=0)	1.168	0.046	0.700	<0.001
(SR) **Iron status**, mg/kg BW				
Iron intake, mg	0.003	0.001	0.078	0.054
Age, years	0.010	0.002	0.199	<0.001
Parity, n	0.048	0.009	0.209	<0.001
Pregnant (=1)	−0.298	0.091	−0.131	0.001
CRP > 5 mg/L	0.034	0.070	0.020	0.625
AGP > 1 g/L	0.093	0.069	0.055	0.175
Chamwino (=1) vs. Kilosa (=0)	0.244	0.045	0.215	<0.001
(LN) **Zinc**, mg/L				
Zinc intake, mg	0.001	0.001	0.015	0.700
Age, years	0.000	0.001	0.023	0.550
Parity, n	−0.002	0.002	−0.035	0.367
Pregnant (=1)	−0.137	0.023	−0.225	<0.001
CRP > 5 mg/L	−0.057	0.019	−0.114	0.003
AGP > 1 g/L	−0.037	0.019	−0.077	0.047
Chamwino (=1) vs. Kilosa (=0)	−0.047	0.012	−0.132	0.001

B, beta coefficient; SE, standard error; Beta, standardized beta coefficient, and *p*, *p*-value of linear regression analysis.
